# Crystal structure of methyl 2-(2*H*-1,3-benzodioxol-5-yl)-7,9-di­bromo-8-oxo-1-oxa­spiro­[4.5]deca-2,6,9-triene-3-car­boxyl­ate

**DOI:** 10.1107/S1600536814024763

**Published:** 2014-11-21

**Authors:** Lucimara Julio Martins, Deborah de Alencar Simoni, Ricardo Aparicio, Fernando Coelho

**Affiliations:** a Laboratory of Synthesis of Natural Products and Drugs, Institute of Chemistry, University of Campinas, PO Box 6154 - 13083-970, Campinas, SP, Brazil; b Laboratory of Single Crystal X-Ray Diffraction, Institute of Chemistry, University of Campinas, PO Box 6154 - 13083-970, Campinas, SP, Brazil; c Laboratory of Structural Biology and Crystallography, Institute of Chemistry, University of Campinas, PO Box 6154 - 13083-970, Campinas, SP, Brazil

**Keywords:** Single-crystal X-ray study, spiro-hexa­dienone structure, Morita–Baylis–Hillman adducts

## Abstract

The title compound, C_18_H_12_Br_2_O_6_, was synthesized from Morita–Baylis–Hillman adducts. It incorporates the bromin­ated spiro-hexa­dienone moiety typically exhibited by compounds of this class that exhibit biological activity. Both the brominated cyclo­hexa­dienone and the central five-membered rings are nearly planar (r.m.s. deviations of 0.044 and 0.016 Å, respectively), being almost perpendicularly oriented [inter­planar angle = 89.47 (5)°]. With respect to the central five-membered ring, the brominated cyclo­hexa­dienone ring, the benzodioxol ring and the carboxyl­ate fragment make C—O—C—C, O—C—C—C and C—C—C—O dihedral angles of −122.11 (8), −27.20 (11) and −8.40 (12)°, respectively. An intra­molecular C—H⋯O hydrogen bond occurs. In the crystal, mol­ecules are linked by non-classical C—H⋯O and C—H⋯Br hydrogen bonds resulting in a molecular packing in which the brominated rings are in a head-to-head orientation, forming well marked planes parallel to the *b* axis.

## Related literature   

For compounds that contain a spiro-hexa­dienone moiety in their structures, related biological activities and examples of brominated spiro-hexa­dienones, see: König & Wright (1993 ▶); Lou (2012 ▶); Sorek *et al.* (2009 ▶). For strategies for the synthesis of spiro-hexa­dienones from Morita–Baylis–Hillman aducts, see: Martins *et al.* (2014 ▶); Barontini *et al.* (2013 ▶).
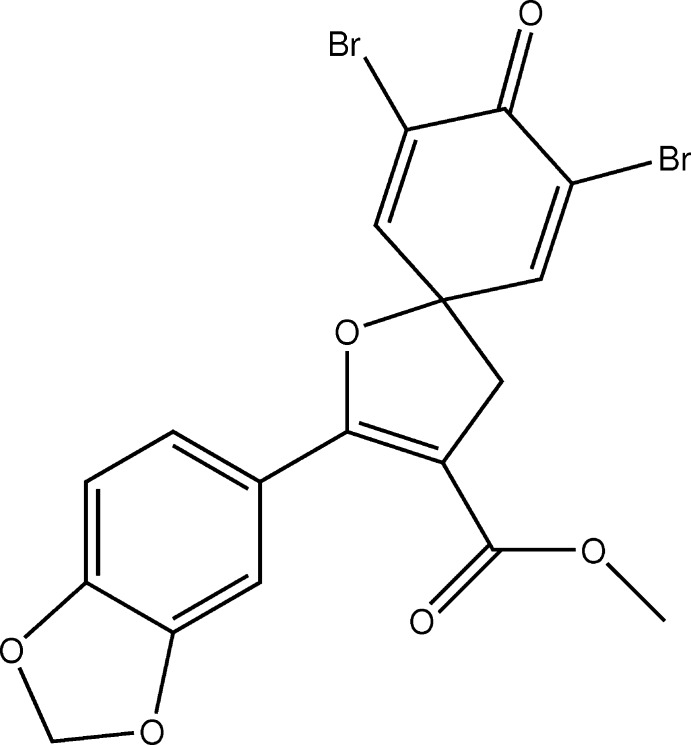


## Experimental   

### Crystal data  

C_18_H_12_Br_2_O_6_
*M* *_r_* = 484.10
Triclinic, 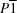

*a* = 8.1929 (13) Å
*b* = 8.4811 (14) Å
*c* = 12.761 (2) Å
α = 84.485 (4)°
β = 80.007 (5)°
γ = 78.077 (4)°
*V* = 852.7 (2) Å^3^
*Z* = 2
Mo *K*α radiation
μ = 4.79 mm^−1^
*T* = 100 K
0.32 × 0.17 × 0.16 mm

### Data collection  

Bruker APEX CCD detector diffractometer
Absorption correction: multi-scan (*SADABS*; Bruker, 2010 ▶) *T*_min_ = 0.309, *T*_max_ = 0.515
27788 measured reflections
7098 independent reflections
6288 reflections with *I* > 2σ(*I*)
*R*_int_ = 0.017

### Refinement  

*R*[*F* ^2^ > 2σ(*F* ^2^)] = 0.018
*wR*(*F* ^2^) = 0.047
*S* = 1.02
7098 reflections
236 parameters
H-atom parameters constrained
Δρ_max_ = 0.57 e Å^−3^
Δρ_min_ = −0.39 e Å^−3^

### 

Data collection: *APEX2* (Bruker, 2010 ▶); cell refinement: *SAINT* (Bruker, 2010 ▶); data reduction: *SAINT*; program(s) used to solve structure: *SHELXS97* (Sheldrick, 2008 ▶); program(s) used to refine structure: *SHELXLE* (Hübschle *et al.*, 2011 ▶) and *SHELXL2014* (Sheldrick, 2008 ▶); molecular graphics: *Mercury* (Macrae *et al.*, 2006 ▶); software used to prepare material for publication: *OLEX2* (Dolomanov *et al.*, 2003 ▶), *PLATON* (Spek, 2009 ▶) and *publCIF* (Westrip, 2010 ▶).

## Supplementary Material

Crystal structure: contains datablock(s) I, New_Global_Publ_Block. DOI: 10.1107/S1600536814024763/hg5413sup1.cif

Structure factors: contains datablock(s) I. DOI: 10.1107/S1600536814024763/hg5413Isup2.hkl

Supporting information file. DOI: 10.1107/S1600536814024763/hg5413Isup4.cdx

Supporting information file. DOI: 10.1107/S1600536814024763/hg5413Isup4.cml

**Fig. 1.**
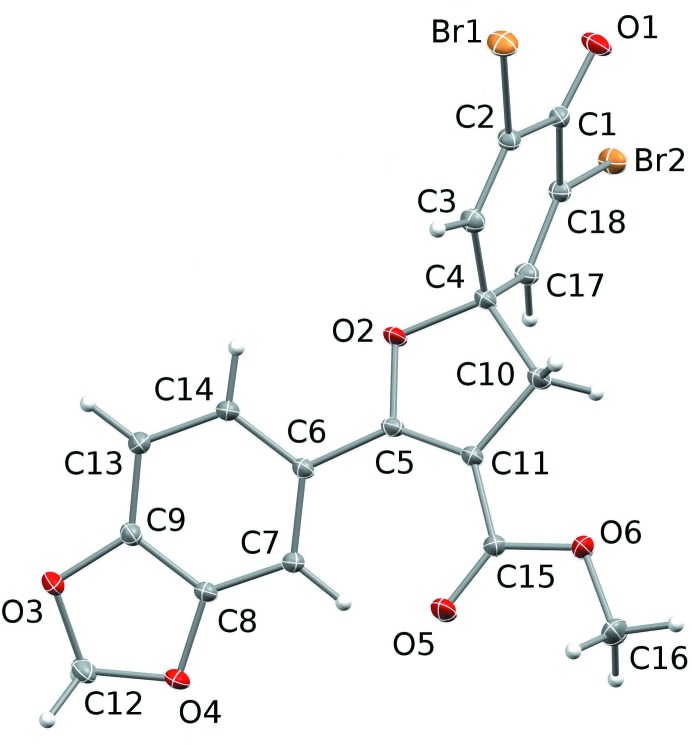
The molecular structure of the title compound with atom labels and 50% probability displacement ellipsoids.

. DOI: 10.1107/S1600536814024763/hg5413fig1.tif
The mol­ecular structure of the title compound with atom labels and 50% probability displacement ellipsoids.

**Fig. 2.**
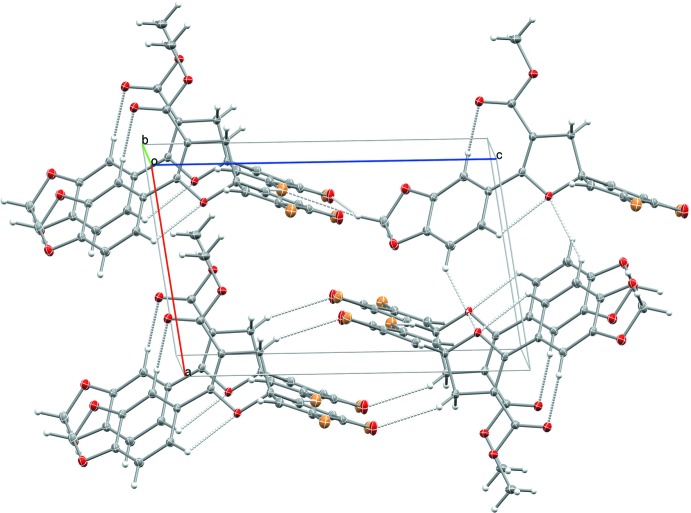
Crystal packing of the title compound, showing hydrogen bonding interactions.

. DOI: 10.1107/S1600536814024763/hg5413fig2.tif
Crystal packing of the title compound, showing hydrogen bonding inter­actions.

CCDC reference: 1033626

Additional supporting information:  crystallographic information; 3D view; checkCIF report

## Figures and Tables

**Table 1:** Hydrogen-bond geometry (, )

*D*H*A*	*D*H	H*A*	*D* *A*	*D*H*A*
C3H3O5^i^	0.95	2.61	3.4272(13)	144
C7H7O5	0.95	2.34	2.9441(13)	121
C10H10*A*O1^ii^	0.99	2.55	3.3995(13)	143
C12H12*A*Br1^iii^	0.99	2.96	3.9411(12)	173
C12H12*A*O1^iii^	0.99	2.53	3.0852(13)	116
C13H13O2^iv^	0.95	2.63	3.3909(13)	137
C16H16*C*O5^v^	0.98	2.59	3.2434(14)	124
C17H17Br1^vi^	0.95	3.03	3.9069(11)	153

Symmetry codes: (i) 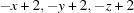; (ii) 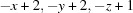; (iii) 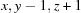; (iv) 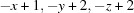; (v) 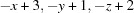; (vi) 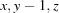.

## supplementary crystallographic information

### S1. Introduction

Compounds containing a spiro-hexadienone moiety typically exhibit biological activity, also sharing a structural architecture observed in some natural products (König & Wright, 1993; Sorek *et al.*, 2009). In view of the importance of this class of compounds, recent efforts resulted in a new synthetic strategy, which starts from Morita-Baylis-Hillman adducts as building blocks for organic synthesis (Martins *et al.*, 2014). Based on this methodology, it was possible to obtain a brominated spiro-hexadienone which resulted, to our knowledge, in the first report of a halogenated spiro-hexadienone crystal structure.

### S2.1. Synthesis and crystallization

Methyl 2-(2H-1,3-benzodioxol-5-yl)-7,9-di­bromo-8-oxo-1-oxa­spiro­[4.5]deca-2,6,9-triene-3-carboxyl­ate was prepared from a subset of β-ketoesters following the experimental protocol recently described by Barontini *et al.* (2013)*.* A separable mixture of mono- and dibrominated (in majority) derivatives in good overall yields was obtained.

After chromatographic separation, the dibrominated compounds were easily transformed into halogenated spiro-hexadienones, in three steps procedure, starting with the Morita-Baylis-Hillman adducts.

Methyl 2-(2H-1,3-benzodioxol-5-yl)-7,9-di­bromo-8-oxo-1-oxa­spiro­[4.5]deca-2,6,9-triene-3-carboxyl­ate (48 mg, 0.1 mmoL) was dissolved in absolute chloro­form-D1 (1 mL), followed by stirring until total dissolution was achieved. The solution was kept in the freezer. After two weeks, the resulting material was filtered under vacuum, washed with small portions of cold chloro­form and dried in a desiccator to furnish pale yellow single crystals suitable for X-ray diffraction data collection.

### S2.2. Refinement

The C-bound H atoms were positioned with idealized geometry and treated as riding atoms: phenyl, methyl and methyl­ene C—H bond lengths were 0.95, 0.98 and 0.99 Å, respectively. The isotropic displacement parameters values (U_iso_(H)) were fixed at 1.5U_eq_(C) for methyl H atoms and 1.2U_eq_(C) for all other attached H atoms.

### S3. Results and discussion

The title compound (Fig. 1) crystallized in the space group P1 assuming a conformational structure determined by non-classical intra­molecular C—H—O and inter­molecular C—H—O and C—H—Br bonding (Table 1, Fig. 2). The molecule contains one six-membered (brominated ciclohexadienone) and one central five-membered rings connected by a spiro-carbon C4. The mean plane of these planar rings, C1—C2—C4—C17—C18 (r.m.s.= 0.044 Å) and C4—C10—C11—C5—O2 (r.m.s. = 0.016 Å), are almost perpendicularly oriented, making a plane-plane angle of 90.53°. With respect to the central five-membered ring, the brominated spiro-hexadienone and the benzodioxol rings make dihedral angles C5—O2—C4—C3 = -122.11 (8)° and O2—C5—C6—C14 = -27.20 (11)°, respectively, while the dihedral angle with the carboxyl­ate fragment C10—C11—C15—O6 is -8.40 (12)°.

In the hexadienone ring of the title compound, the C2—C3, C17—C18 and C1—O1 bond lengths are 1.3351 (13), 1.3354 (13) and 1.2139 (11) Å, respectively, and the bond length between the spiro-carbon (C4) and the oxygen atom C4—O2 is 1.4684 (11) Å, similar to those reported for a related oxa­spiro structure (Lou, 2012). In the latter, the plane-plane angle between the mean planes of the six-membered and the central five-membered rings in the spiro-carbon is 96.06°, slightly different from that observed in the title compound (90.53°). Further comparison also reveals different orientations of the carboxyl­ate moiety, which makes a dihedral angle of 170.6 (1)° (C10—C11—C15—O5) in the title compound, with a corresponding angle equal to 11.3 (3)° in the related structure (Lou, 2012). This large difference is consistent with an observed C7—H7—O5 intra­molecular hydrogen bond (Table 1) in the title compound, which results in a favourable conformation of the carboxyl­ate group.

### Crystal data

**Table:**

C_18_H_12_Br_2_O_6_	*Z* = 2
*M**_r_* = 484.10	*F*(000) = 476
Triclinic, *P*1	*D*_x_ = 1.885 Mg m^−^^3^
Hall symbol: -P 1	Mo *K*α radiation, λ = 0.71073 Å
*a* = 8.1929 (13) Å	Cell parameters from 9887 reflections
*b* = 8.4811 (14) Å	θ = 3.2–35.1°
*c* = 12.761 (2) Å	µ = 4.79 mm^−^^1^
α = 84.485 (4)°	*T* = 100 K
β = 80.007 (5)°	Block, pale yellow
γ = 78.077 (4)°	0.32 × 0.17 × 0.16 mm
*V* = 852.7 (2) Å^3^	

### Data collection

**Table:**

Bruker APEX CCD detector diffractometer	7098 independent reflections
Radiation source: fine-focus sealed tube	6288 reflections with *I* > 2σ(*I*)
Detector resolution: 8.3333 pixels mm^-1^	*R*_int_ = 0.017
phi and ω scans	θ_max_ = 34.3°, θ_min_ = 3.0°
Absorption correction: multi-scan (*SADABS*; Bruker, 2010)	*h* = −12→12
*T*_min_ = 0.309, *T*_max_ = 0.515	*k* = −13→13
27788 measured reflections	*l* = −20→20

### Refinement

**Table:**

Refinement on *F*^2^	0 restraints
Least-squares matrix: full	Hydrogen site location: inferred from neighbouring sites
*R*[*F*^2^ > 2σ(*F*^2^)] = 0.018	H-atom parameters constrained
*wR*(*F*^2^) = 0.047	*w* = 1/[σ^2^(*F*_o_^2^) + (0.0245*P*)^2^ + 0.2583*P*] where *P* = (*F*_o_^2^ + 2*F*_c_^2^)/3
*S* = 1.02	(Δ/σ)_max_ = 0.003
7098 reflections	Δρ_max_ = 0.57 e Å^−^^3^
236 parameters	Δρ_min_ = −0.39 e Å^−^^3^

### Special details

**Table:**

Geometry. All e.s.d.'s (except the e.s.d. in the dihedral angle between two l.s. planes) are estimated using the full covariance matrix. The cell e.s.d.'s are taken into account individually in the estimation of e.s.d.'s in distances, angles and torsion angles; correlations between e.s.d.'s in cell parameters are only used when they are defined by crystal symmetry. An approximate (isotropic) treatment of cell e.s.d.'s is used for estimating e.s.d.'s involving l.s. planes.

### Fractional atomic coordinates and isotropic or equivalent isotropic displacement parameters (Å^2^)

**Table:**

	*x*	*y*	*z*	*U*_iso_*/*U*_eq_	
Br1	0.81611 (2)	1.44904 (2)	0.62579 (2)	0.01782 (3)	
Br2	0.72560 (2)	0.85491 (2)	0.49155 (2)	0.01638 (3)	
O1	0.75814 (11)	1.20677 (9)	0.48397 (6)	0.01915 (14)	
O2	0.81234 (9)	0.93071 (9)	0.86474 (5)	0.01450 (12)	
O3	0.57197 (10)	0.68379 (9)	1.33065 (6)	0.01608 (13)	
O4	0.81596 (9)	0.50402 (9)	1.26861 (6)	0.01609 (13)	
O5	1.24024 (10)	0.67487 (10)	1.01728 (6)	0.01916 (14)	
O6	1.37041 (9)	0.72220 (9)	0.84968 (6)	0.01529 (12)	
C1	0.79228 (12)	1.13898 (10)	0.56785 (7)	0.01214 (14)	
C2	0.83286 (12)	1.22429 (10)	0.65381 (7)	0.01222 (14)	
C3	0.88062 (12)	1.14902 (11)	0.74351 (7)	0.01357 (15)	
H3	0.8963	1.2114	0.7977	0.016*	
C4	0.91021 (12)	0.96878 (11)	0.76100 (7)	0.01264 (15)	
C5	0.91926 (12)	0.83972 (10)	0.92957 (7)	0.01135 (14)	
C6	0.82769 (11)	0.79788 (10)	1.03513 (7)	0.01106 (14)	
C7	0.88494 (12)	0.65586 (11)	1.09690 (7)	0.01202 (14)	
H7	0.9864	0.5828	1.0731	0.014*	
C8	0.78592 (12)	0.62914 (11)	1.19325 (7)	0.01182 (14)	
C9	0.63896 (12)	0.73620 (11)	1.23042 (7)	0.01254 (14)	
C10	1.09810 (12)	0.89627 (12)	0.77135 (7)	0.01504 (16)	
H10A	1.1501	0.8183	0.7164	0.018*	
H10B	1.1655	0.9820	0.7656	0.018*	
C11	1.08297 (12)	0.81321 (11)	0.88176 (7)	0.01190 (14)	
C12	0.66357 (14)	0.52054 (12)	1.34563 (8)	0.01708 (17)	
H12A	0.6912	0.5003	1.4188	0.020*	
H12B	0.5948	0.4422	1.3344	0.020*	
C13	0.58111 (12)	0.87530 (11)	1.17181 (8)	0.01435 (15)	
H13	0.4809	0.9486	1.1975	0.017*	
C14	0.67762 (12)	0.90331 (11)	1.07223 (7)	0.01288 (15)	
H14	0.6401	0.9965	1.0287	0.015*	
C15	1.23289 (12)	0.72953 (11)	0.92621 (7)	0.01239 (14)	
C16	1.52864 (13)	0.64225 (14)	0.88280 (9)	0.02125 (19)	
H16A	1.5418	0.6880	0.9478	0.032*	
H16B	1.6222	0.6580	0.8261	0.032*	
H16C	1.5291	0.5265	0.8969	0.032*	
C17	0.85126 (12)	0.88535 (11)	0.68028 (7)	0.01319 (15)	
H17	0.8524	0.7728	0.6918	0.016*	
C18	0.79718 (12)	0.96291 (10)	0.59287 (7)	0.01170 (14)	

### Atomic displacement parameters (Å^2^)

**Table:**

	*U*^11^	*U*^22^	*U*^33^	*U*^12^	*U*^13^	*U*^23^
Br1	0.02471 (5)	0.00997 (4)	0.01954 (5)	−0.00466 (3)	−0.00492 (4)	0.00072 (3)
Br2	0.02188 (5)	0.01408 (4)	0.01473 (4)	−0.00300 (3)	−0.00654 (3)	−0.00306 (3)
O1	0.0307 (4)	0.0153 (3)	0.0107 (3)	−0.0017 (3)	−0.0063 (3)	0.0023 (2)
O2	0.0119 (3)	0.0197 (3)	0.0100 (3)	−0.0004 (2)	−0.0030 (2)	0.0048 (2)
O3	0.0186 (3)	0.0160 (3)	0.0112 (3)	−0.0020 (2)	0.0011 (2)	0.0023 (2)
O4	0.0173 (3)	0.0147 (3)	0.0138 (3)	−0.0009 (2)	−0.0019 (2)	0.0056 (2)
O5	0.0146 (3)	0.0278 (4)	0.0125 (3)	0.0000 (3)	−0.0034 (2)	0.0045 (3)
O6	0.0106 (3)	0.0196 (3)	0.0133 (3)	0.0005 (2)	−0.0009 (2)	0.0011 (2)
C1	0.0137 (4)	0.0115 (3)	0.0101 (3)	−0.0011 (3)	−0.0008 (3)	−0.0001 (3)
C2	0.0144 (4)	0.0101 (3)	0.0119 (4)	−0.0024 (3)	−0.0017 (3)	0.0000 (3)
C3	0.0153 (4)	0.0137 (3)	0.0123 (4)	−0.0032 (3)	−0.0034 (3)	−0.0009 (3)
C4	0.0137 (4)	0.0143 (3)	0.0091 (3)	−0.0018 (3)	−0.0025 (3)	0.0023 (3)
C5	0.0129 (4)	0.0113 (3)	0.0096 (3)	−0.0011 (3)	−0.0037 (3)	0.0010 (3)
C6	0.0123 (4)	0.0117 (3)	0.0093 (3)	−0.0021 (3)	−0.0028 (3)	0.0004 (3)
C7	0.0128 (4)	0.0113 (3)	0.0114 (3)	−0.0009 (3)	−0.0024 (3)	0.0003 (3)
C8	0.0135 (4)	0.0111 (3)	0.0109 (3)	−0.0020 (3)	−0.0036 (3)	0.0012 (3)
C9	0.0139 (4)	0.0133 (3)	0.0101 (3)	−0.0028 (3)	−0.0012 (3)	−0.0001 (3)
C10	0.0131 (4)	0.0196 (4)	0.0110 (4)	−0.0014 (3)	−0.0027 (3)	0.0036 (3)
C11	0.0122 (4)	0.0133 (3)	0.0095 (3)	−0.0010 (3)	−0.0024 (3)	0.0009 (3)
C12	0.0219 (5)	0.0149 (4)	0.0127 (4)	−0.0037 (3)	0.0000 (3)	0.0027 (3)
C13	0.0139 (4)	0.0140 (4)	0.0130 (4)	0.0005 (3)	−0.0006 (3)	0.0004 (3)
C14	0.0131 (4)	0.0127 (3)	0.0116 (4)	−0.0004 (3)	−0.0026 (3)	0.0015 (3)
C15	0.0115 (4)	0.0132 (3)	0.0117 (4)	−0.0005 (3)	−0.0023 (3)	−0.0003 (3)
C16	0.0119 (4)	0.0287 (5)	0.0201 (5)	0.0015 (4)	−0.0019 (3)	0.0012 (4)
C17	0.0151 (4)	0.0114 (3)	0.0126 (4)	−0.0018 (3)	−0.0031 (3)	0.0012 (3)
C18	0.0134 (4)	0.0110 (3)	0.0108 (3)	−0.0018 (3)	−0.0025 (3)	−0.0011 (3)

### Geometric parameters (Å, º)

**Table:**

Br1—C2	1.8863 (9)	C6—C14	1.4001 (13)
Br2—C18	1.8887 (9)	C6—C7	1.4158 (12)
O1—C1	1.2139 (11)	C7—C8	1.3775 (13)
O2—C5	1.3772 (11)	C7—H7	0.9500
O2—C4	1.4684 (11)	C8—C9	1.3900 (13)
O3—C9	1.3721 (11)	C9—C13	1.3777 (13)
O3—C12	1.4444 (12)	C10—C11	1.5104 (13)
O4—C8	1.3746 (11)	C10—H10A	0.9900
O4—C12	1.4397 (13)	C10—H10B	0.9900
O5—C15	1.2158 (11)	C11—C15	1.4623 (13)
O6—C15	1.3520 (11)	C12—H12A	0.9900
O6—C16	1.4459 (13)	C12—H12B	0.9900
C1—C2	1.4887 (13)	C13—C14	1.4026 (13)
C1—C18	1.4904 (12)	C13—H13	0.9500
C2—C3	1.3351 (13)	C14—H14	0.9500
C3—C4	1.4985 (13)	C16—H16A	0.9800
C3—H3	0.9500	C16—H16B	0.9800
C4—C17	1.4996 (13)	C16—H16C	0.9800
C4—C10	1.5585 (14)	C17—C18	1.3354 (13)
C5—C11	1.3563 (13)	C17—H17	0.9500
C5—C6	1.4733 (12)		
			
C5—O2—C4	109.46 (7)	C11—C10—H10A	111.3
C9—O3—C12	104.80 (7)	C4—C10—H10A	111.3
C8—O4—C12	105.13 (7)	C11—C10—H10B	111.3
C15—O6—C16	115.44 (8)	C4—C10—H10B	111.3
O1—C1—C2	122.93 (8)	H10A—C10—H10B	109.2
O1—C1—C18	122.50 (8)	C5—C11—C15	128.64 (8)
C2—C1—C18	114.57 (8)	C5—C11—C10	110.13 (8)
C3—C2—C1	123.31 (8)	C15—C11—C10	121.07 (8)
C3—C2—Br1	121.81 (7)	O4—C12—O3	106.94 (7)
C1—C2—Br1	114.87 (6)	O4—C12—H12A	110.3
C2—C3—C4	121.73 (8)	O3—C12—H12A	110.3
C2—C3—H3	119.1	O4—C12—H12B	110.3
C4—C3—H3	119.1	O3—C12—H12B	110.3
O2—C4—C3	107.32 (7)	H12A—C12—H12B	108.6
O2—C4—C17	106.47 (7)	C9—C13—C14	116.62 (9)
C3—C4—C17	114.33 (8)	C9—C13—H13	121.7
O2—C4—C10	105.43 (7)	C14—C13—H13	121.7
C3—C4—C10	111.56 (8)	C6—C14—C13	121.91 (8)
C17—C4—C10	111.13 (8)	C6—C14—H14	119.0
C11—C5—O2	112.55 (8)	C13—C14—H14	119.0
C11—C5—C6	135.58 (8)	O5—C15—O6	122.92 (9)
O2—C5—C6	111.85 (8)	O5—C15—C11	127.60 (9)
C14—C6—C7	120.45 (8)	O6—C15—C11	109.47 (8)
C14—C6—C5	117.27 (8)	O6—C16—H16A	109.5
C7—C6—C5	122.27 (8)	O6—C16—H16B	109.5
C8—C7—C6	116.53 (8)	H16A—C16—H16B	109.5
C8—C7—H7	121.7	O6—C16—H16C	109.5
C6—C7—H7	121.7	H16A—C16—H16C	109.5
O4—C8—C7	127.79 (8)	H16B—C16—H16C	109.5
O4—C8—C9	109.53 (8)	C18—C17—C4	122.64 (8)
C7—C8—C9	122.61 (8)	C18—C17—H17	118.7
O3—C9—C13	128.16 (9)	C4—C17—H17	118.7
O3—C9—C8	109.94 (8)	C17—C18—C1	122.38 (8)
C13—C9—C8	121.85 (8)	C17—C18—Br2	121.91 (7)
C11—C10—C4	102.30 (7)	C1—C18—Br2	115.70 (6)

### Hydrogen-bond geometry (Å, º)

**Table:**

*D*—H···*A*	*D*—H	H···*A*	*D*···*A*	*D*—H···*A*
C3—H3···O5^i^	0.95	2.61	3.4272 (13)	144
C7—H7···O5	0.95	2.34	2.9441 (13)	121
C10—H10*A*···O1^ii^	0.99	2.55	3.3995 (13)	143
C12—H12*A*···Br1^iii^	0.99	2.96	3.9411 (12)	173
C12—H12*A*···O1^iii^	0.99	2.53	3.0852 (13)	116
C13—H13···O2^iv^	0.95	2.63	3.3909 (13)	137
C16—H16*C*···O5^v^	0.98	2.59	3.2434 (14)	124
C17—H17···Br1^vi^	0.95	3.03	3.9069 (11)	153
C14—H14···O2	0.95	2.36	2.6937 (12)	100

Symmetry codes: (i) −*x*+2, −*y*+2, −*z*+2; (ii) −*x*+2, −*y*+2, −*z*+1; (iii) *x*, *y*−1, *z*+1; (iv) −*x*+1, −*y*+2, −*z*+2; (v) −*x*+3, −*y*+1, −*z*+2; (vi) *x*, *y*−1, *z*.
