# Supplementary figures and images for: Crystal structure of methyl 2-(2H-1,3-benzodioxol-5-yl)-7,9-di­bromo-8-oxo-1-oxa­spiro­[4.5]deca-2,6,9-triene-3-car­boxyl­ate
Source: Acta Crystallogr Sect E Struct Rep Online. 2014 Nov 21;70(Pt 12):o1275–6. doi: 10.1107/S1600536814024763 (PMC4257376; doi:10.1107/S1600536814024763)

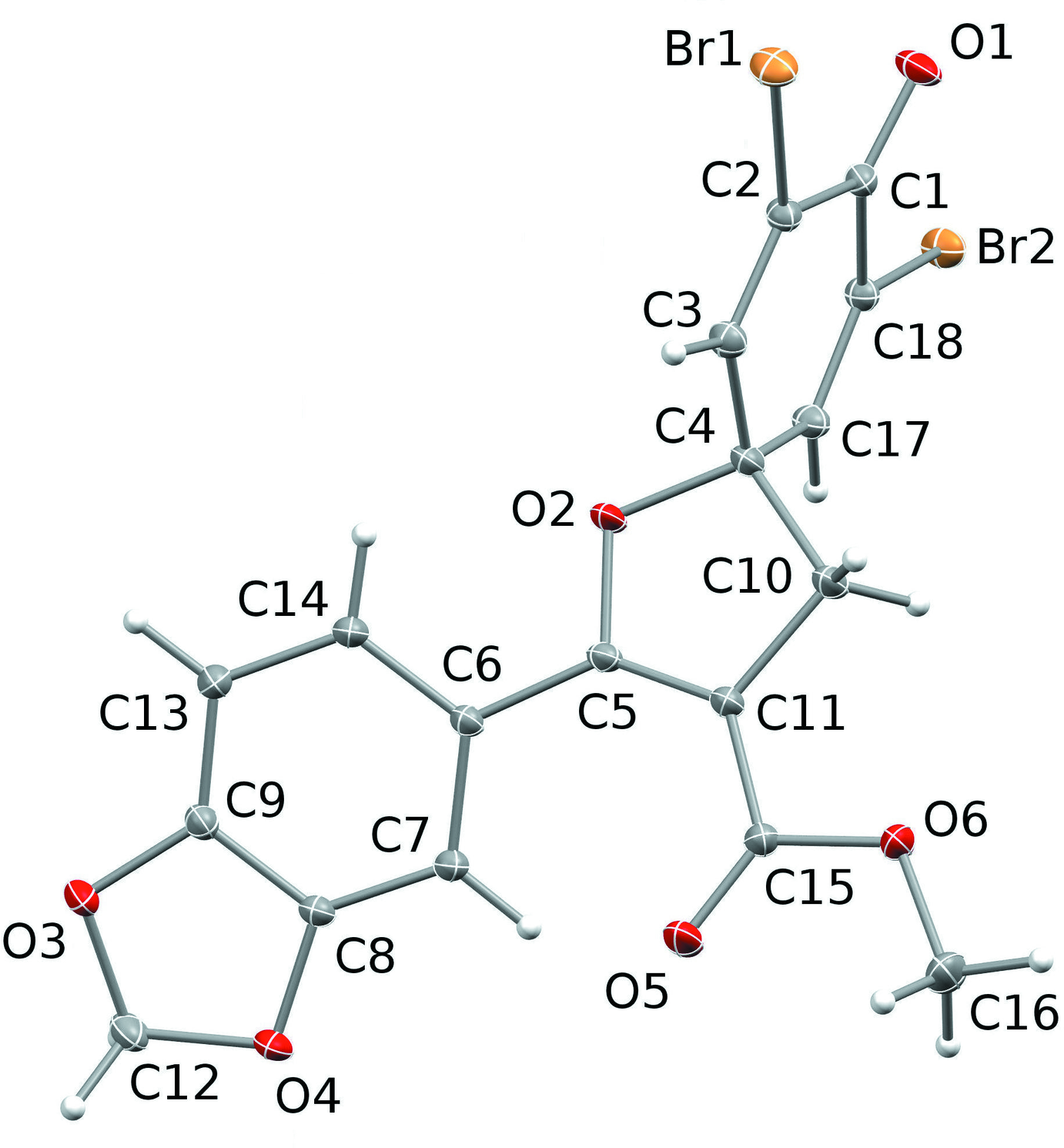

Supplement: Supplementary file 5 [file e-70-o1275-fig1.tif]

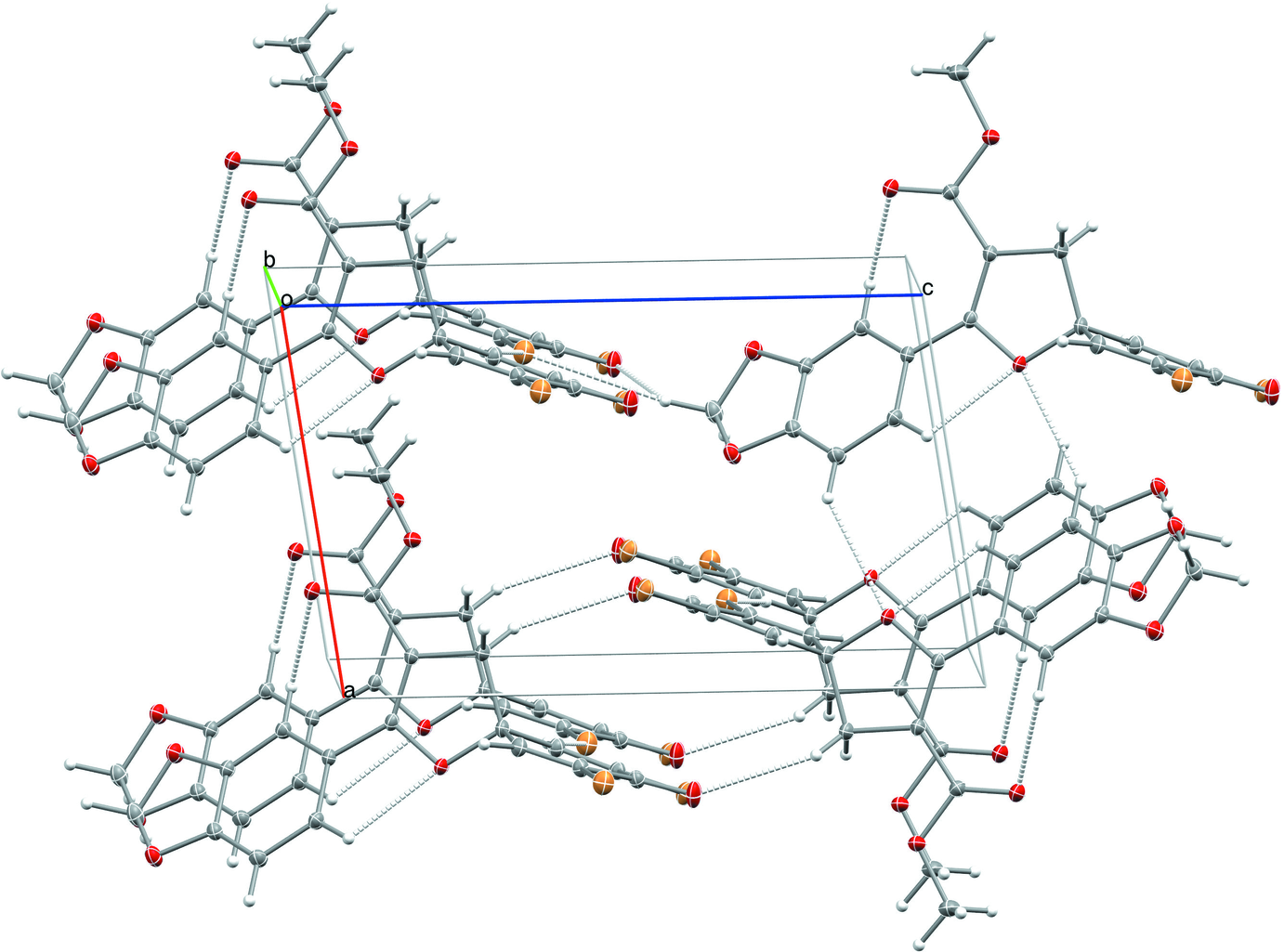

Supplement: Supplementary file 6 [file e-70-o1275-fig2.tif]
